# Domain 4 (D4) of Perfringolysin O to Visualize Cholesterol in Cellular Membranes—The Update

**DOI:** 10.3390/s17030504

**Published:** 2017-03-03

**Authors:** Masashi Maekawa

**Affiliations:** 1 Department of Biochemistry and Molecular Genetics, Ehime University Graduate School of Medicine, Toon, Ehime 791-0295, Japan; masashim@m.ehime-u.ac.jp; Tel.: +81-89-960-5254; 2 Division of Cell Growth and Tumor Regulation, Proteo-Science Center, Ehime University; Toon, Ehime 791-0295, Japan

**Keywords:** cholesterol probes, visualization, domain 4, D4, Perfringolysin O, theta toxin, microscopy

## Abstract

The cellular membrane of eukaryotes consists of phospholipids, sphingolipids, cholesterol and membrane proteins. Among them, cholesterol is crucial for various cellular events (e.g., signaling, viral/bacterial infection, and membrane trafficking) in addition to its essential role as an ingredient of steroid hormones, vitamin D, and bile acids. From a micro-perspective, at the plasma membrane, recent emerging evidence strongly suggests the existence of lipid nanodomains formed with cholesterol and phospholipids (e.g., sphingomyelin, phosphatidylserine). Thus, it is important to elucidate how cholesterol behaves in membranes and how the behavior of cholesterol is regulated at the molecular level. To elucidate the complexed characteristics of cholesterol in cellular membranes, a couple of useful biosensors that enable us to visualize cholesterol in cellular membranes have been recently developed by utilizing domain 4 (D4) of Perfringolysin O (PFO, theta toxin), a cholesterol-binding toxin. This review highlights the current progress on development of novel cholesterol biosensors that uncover new insights of cholesterol in cellular membranes.

## 1. Introduction

Cells produce cholesterol through the de novo pathway in the endoplasmic reticulum (ER) and this lipid is transported to cellular compartments via either vesicular or non-vesicular routes [1]. A part of cholesterol is esterified with fatty acids by acyl-CoA cholesterol acyl transferase (ACAT) in ER and the esterified cholesterol is stored in lipid droplets (LDs) [1]. In addition, cells get cholesterol exogenously by the uptake of some lipoproteins, mainly low-density lipoprotein (LDL) [1]. LDL internalized via the LDL receptor is transported to the multivesicular bodies (MVBs) through the endosomal pathway and degraded to release non-esterified cholesterol [1]. Cholesterol derived from LDL utilizes two late endosomal proteins, NPC1 and NPC2, to escape from lumen of the late endosomes (LEs) and then cholesterol is delivered to other cellular membranes [1,2,3]. Critically, cholesterol is heterogeneously distributed in animal cells. Cholesterol is enriched in the plasma membrane (PM) and transferrin receptor-positive recycling endosomes (REs), whereas content of cholesterol in the ER maintains low levels [1,4,5]. The precise mechanisms for determination of this heterogeneous cellular distribution of cholesterol is still unclear, although some lipid transfer proteins (e.g., OSBP, START domain proteins) and membrane-trafficking-related proteins (e.g., Rab11, Rab8) contribute to proper cellular distribution of cholesterol [6,7,8,9,10].

**Figure 1:**
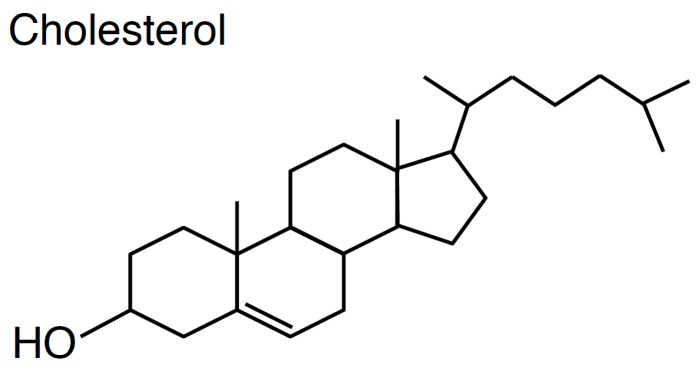
Molecular structure of cholesterol.

One of the hallmarks of cholesterol is that this lipid has a rigid four-ring sterol backbone that is hydrophobic and possesses only a hydroxyl group as a polar head group (Figure 1). The large portion of hydrophobicity of cholesterol decides its orientation in the phospholipids bilayer that cholesterol is located parallel to fatty acyl chains of phospholipids [11]. Owing to the small hydrophilic group of cholesterol, unlike phospholipids and sphingolipids, cholesterol exhibits very fast spontaneous transbilayer movement (flip-flop) [12,13]. Critically, the small hydroxyl group of cholesterol is insufficient to shield the comparatively large hydrophobic group of cholesterol from water molecules in the lipid bilayer. Thus, it is widely believed that cholesterol in the lipid bilayer prefers to interact with phospholipids containing large polar head groups and long saturated fatty acyl chains like sphingomyelin [1,14,15,16]. This concept has generated a notion that cholesterol and sphingolipids form nanodomains in the exofacial leaflets of the PM, called lipid rafts, and this theory has been proven by a variety of biochemical and microscopic experiments [17,18,19,20]. However, it remains unclear if other cholesterol nanodomains exist in cellular membranes, especially in the cytosolic leaflets of the PM (see Section 2.4). In addition, the distribution of cholesterol between the exofacial leaflets and cytosolic leaflets of the PM is still debatable (see Section 2.5). From the patho-physiological standpoint, numerous studies have shown that cholesterol in the PM has crucial functions in cell signalling, endocytosis, bacterial/viral infection, cancer, angiogenesis, and Alzheimer’s disease [21,22,23,24,25,26]. Furthermore, cholesterol regulates the recycling pathway, which is consistent with enrichment of cholesterol in REs [5,27,28,29,30,31,32].

**Figure 2:**
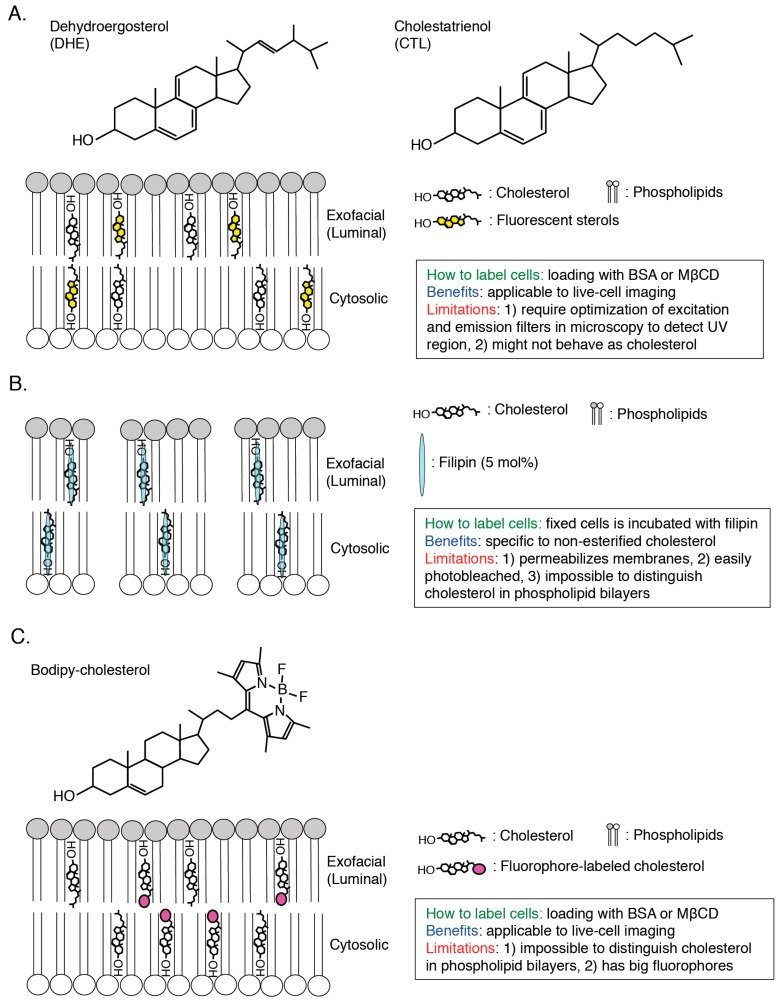
Scheme of visualization of cholesterol in cellular membranes by fluorescent sterols (**A**) filipin (**B**) and fluorophore-labelled cholesterol (**C**) Methodology, benefits and limitations of each cholesterol biosensor are described. Threshold for cholesterol-binding to filipin, 5 mol %, is shown ([37]; **B**).

**Figure 3:**
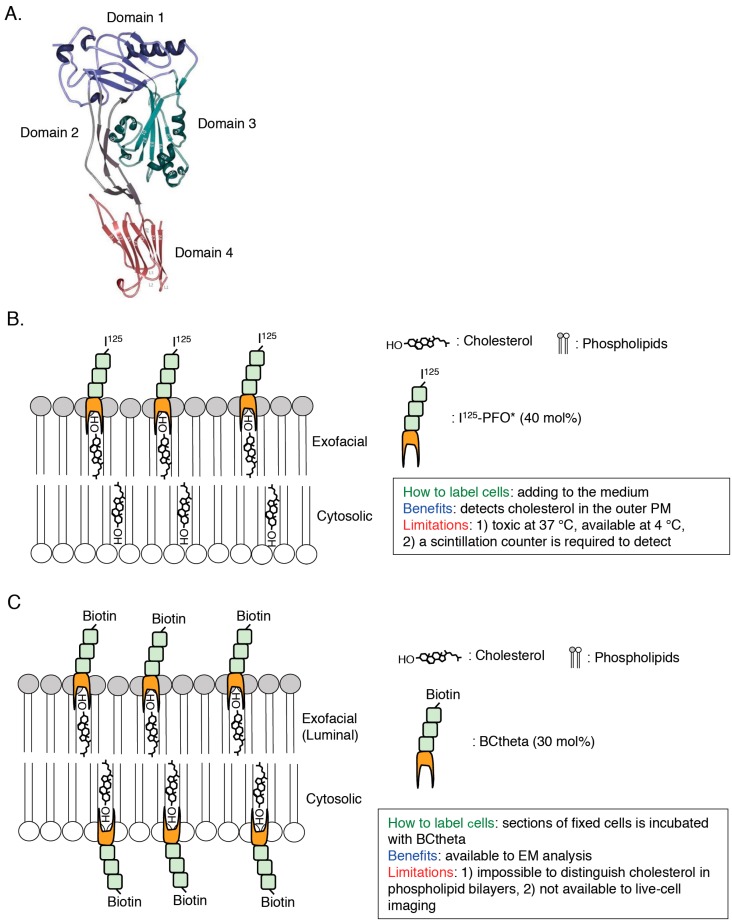
Scheme of detection of cholesterol in cellular membranes by PFO-derived cholesterol biosensors. (**A**) Domain structure of PFO. The image is reproduced from [62]. Copyright MDPI 2015. (**B**,**C**) Scheme, methodology, benefits, and limitations of I^125^-PFO* (**B**) and BCtheta (**C**) are shown. The number is cholesterol concentration (mol %) threshold required for each cholesterol probe-binding.

**Figure 4:**
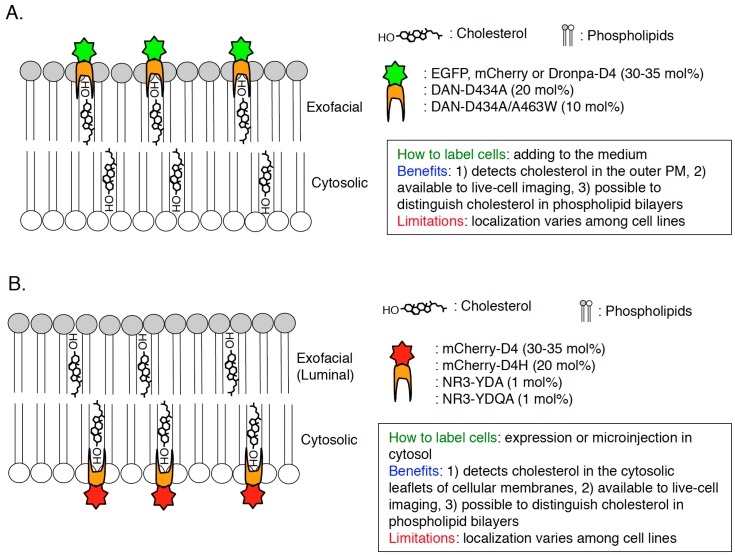
Scheme of visualization of cholesterol in cellular membranes by D4-derived cholesterol biosensors. Scheme, methodology, benefits, and limitations of D4-derived cholesterol biosensors to visualize cholesterol in the exofacial leaflets of the PM (**A**) and cytosolic leaflets of cellular membranes (**B**) are shown. The number is cholesterol concentration (mol %) threshold required for each cholesterol probe-binding.

To elucidate the characteristics of cholesterol in cellular membrane in great details, visualization of cholesterol in a single cell is required and, until now, a variety of cholesterol biosensors have been developed (Figure 2, Figure 3 and Figure 4). Dehydroergosterol (DHE) and cholestatrienol (CTL) are intrinsic fluorescent cholesterol analogues and applicable to live-cell imaging (Figure 2A). Exogenously-loaded DHE was accumulated in the PM and REs in CHO cells and the majority of DHE was shown to localize in the cytosolic leaflets of the PM by quenching experiments [33]. Attention should be paid that these fluorescent cholesterol analogs are not native cholesterol although CTL basically showed indistinguishable characteristics from native cholesterol [34]. Filipin, an intrinsically fluorescent polyene antibiotic, can bind to non-esterified cholesterol specifically and has been widely used for visualization of cholesterol in all of the intracellular membranes ([35,36]; Figure 2B). However, filipin is not available to live-cell imaging because this dye itself has a membrane permeability activity, thus filipin requires fixation to stain cellular cholesterol ([37]; Figure 2B). In addition, another problem is that filipin is easily photo-bleached [38]. Fluorophore-labeled cholesterols (e.g., BODIPY-cholesterol) can be easily loaded in cells with BSA and mainly distribute to endosomes ([39,40,41,42]; Figure 2C). Although these probes can be used for live-cell imaging, addition of fluorophores to cholesterol might change characteristics of cholesterol, thus distribution and dynamics of fluorophore-labeled cholesterols might not always exhibit the same characteristics of endogenous cholesterol [43]. Moreover, one big issue is that filipin and fluorophore-labeled cholesterols cannot distinguish cholesterol in the cytosolic leaflets and luminal (exofacial) leaflets of cellular membranes ([38]; Figure 2). Perfringolysin O (PFO, theta toxin) is a protein secreted by the Gram-positive anaerobe *Clostridium perfringens* and binds to cholesterol in the exofacial leaflets of the PM resulting in pore formation and cell lysis [44]. PFO structurally possesses four domains and, among them, domain 4 (D4) recognizes the hydroxyl group at the position 3 of cholesterol ([45,46]; Figure 1 and Figure 3A). D4 was shown to be sufficient for binding of PFO to cholesterol [47,48]. By introducing a variety of mutations into PFO or D4, which removes its cytotoxicity or increase the affinity to cholesterol, researchers have successfully developed cholesterol biosensors that can distinguish transbilayer distribution of cholesterol in cellular membranes ([44]; Figure 3 and Figure 4). This review will focus on PFO-derived cholesterol biosensors, especially D4 mutants, and show the latest findings, which will open new doors for novel cholesterol biology.

## 2. Cholesterol Biosensors Derived from Perfringolysin O Theta Toxin

### 2.1. PFO*

PFO*, a non-toxic PFO, contains Y181A and C459A mutations [49,50,51,52]. Although fully-active PFO binds to cholesterol in the exofacial leaflets of the PM and induces membrane permeabilization at both 37 °C and 4 °C, PFO* can bind to cholesterol in the exofacial leaflets of the PM without membrane permeabilization at 4 °C [51]. Cholesterol concentration threshold required for PFO*-binding is 40 mol % [51]. This property of PFO* enables us to monitor cholesterol in the exofacial leaflets of the PM by measuring radioactivity of radioisotope-labeled PFO* (^125^I-PFO*) using a scintillation counter ([51]; Figure 3B). This probe clearly showed that cholesterol derived from exogenously internalized LDL is transported from the lysosome to the PM [51]. A recent study using PFO* suggests the existence of three types of pools of cholesterol in the PM, PFO-accessible cholesterol, SM-sequestered cholesterol, and essential cholesterol [53]. PFO* is useful to monitor cholesterol in the exofacial leaflets of the PM. However, this probe is not suitable to live-cell imaging because PFO* permeabilizes the PM at 37 °C [51]. Thus, we cannot obtain enough information of intracellular distribution of cholesterol.

### 2.2. BCtheta

To visualize the cellular distribution of cholesterol, the biotinylated PFO segment, called BCtheta, was developed ([54]; Figure 3C). Subtilisin A cleaved between 144 and 145 amino acids residues of fully-active PFO and this protease-nicked PFO, called Ctheta, can bind to cholesterol without cytotoxicity [55]. BCtheta then was generated by biotinylation of methylated Ctheta [54]. Cholesterol concentration threshold required for BCtheta-binding is approximately 30 mol % [56,57]. Combination of BCtheta and immunoelectron microscopy provides subcellular distribution of cholesterol in great detail [58]. After fixation of the human lymphoblastoid cells with paraformaldehyde, cryosections were prepared followed by labeling the section with BCtheta [58]. BCtheta in the sections was visualized with anti-biotin antibody/protein gold A or avidin-gold conjugate using electron microscopy (EM), and the images clearly showed that BCtheta accumulated in internal membranes of MVBs, exosomes, and REs [58,59]. Although, this EM analysis using BCtheta is a robust method to observe the distribution of cholesterol in cells, it is impossible to observe cholesterol dynamics in living cells. Also, it is noted that both PFO* and BCtheta are comparatively large (53 kDa). These large probes might inhibit binding of these probes to closely-localized cholesterol each other in cellular membranes. Importantly, previous studies have indicated that surrounding environments of cholesterol in model membranes critically affect the binding of PFO to cholesterol (e.g., pH, polar head group of phospholipids, fatty acyl chain composition of phospholipids) [50,51,60,61]. Taken together, improved cholesterol biosensors, which have a smaller size and are less affected by their neighborhood, have been required.

### 2.3. D4

**Figure 5:**
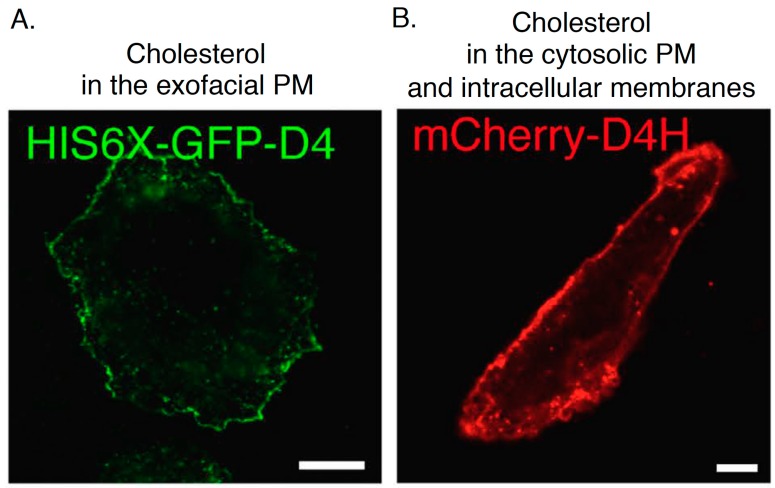
Confocal images of CHO cells labeled with His-GFP-D4 recombinant proteins (**A**) and expressing mCherry-D4H (**B**). Bar, 10 µm. The images are reproduced from [67]. Copyright Nature Publishing Group 2016.

Domain 4 (D4) is a domain of PFO which size is 13 kDa and D4 is the smallest segment that is sufficient to bind to cholesterol with no cytotoxicity ([46,48,63,64]; Figure 3A). D4 associates with cholesterol in membranes through the tip of the four connecting loops and the rest of the domain does not have any contacts with the membrane [48]. In the recent studies, D4 and its mutants are widely used to visualize cholesterol in cellular membranes. Recombinant EGFP or mCherry-D4 proteins can visualize cholesterol in the exofacial leaflets of the PM in the living cells ([65,66,67,68,69]; Figure 4A and Figure 5A). Binding of EGFP-D4 to the exofacial leaflets of the PM can be easily quantified using a flow cytometry [66,67], suggesting that labeling of cholesterol in the outer PM by EGFP-D4 would be suitable for high-throughput screen to identify compounds and genes, which regulate plasmalemmal localization of cholesterol in the exofacial leaflets. By labeling cholesterol in the exofacial leaflets of the PM with Dronpa-D4, cholesterol clusters in the outer PM were visualized using photoactivation localization microscopy (PALM) [70]. In addition to labeling in exofacial cholesterol in the PM, D4 is useful to visualize cholesterol in the cytosolic leaflets of cellular membranes by expression of mCherry-tagged D4 in cytosol in a couple of cell lines ([69,71]; Figure 4B). It should be emphasized that this method can distinguish transbilayer distribution of cholesterol in the PM, by addition of recombinant fluorophore-labeled D4 proteins in the medium and expression of fluorophore-labeled D4 in cytosol to visualize cholesterol in the exofacial and cytosolic leaflets of the PM, respectively ([66,69]; Figure 4). One issue to be considered is that D4 cannot always visualize cholesterol in the exofacial or/and cytosolic leaflets of the PM in a variety of mammalian cells because transbilayer distribution of cholesterol and its content in each leaflet of the PM seems to vary among cell lines. For example, binding of recombinant EGFP-D4 to the exofacial PM is high in Chinese hamster ovary (CHO) cells but very low in Raw cells ([66]; unpublished data). Cytosolically-expressed D4 localizes at the inner PM not in CHO cells but in HeLa and MA-10 cells [69,71]. D4 can bind to liposomes in which cholesterol concentration is more than 30–35 mol % [66,68]. It is likely that cholesterol concentration in the cellular membranes which D4 cannot bind to is lower than 30 mol % and/or environments around cholesterol like phospholipids composition inhibit binding of D4 to cholesterol in the membranes. To solve this issue, a couple of novel and excellent cholesterol probes derived from D4 have been developed by introducing a variety of mutations which decrease the threshold for cholesterol-binding to D4 [66,72].

### 2.4. D4H

A previous study found that a point mutation, D434S in domain 4 decreases the threshold for cholesterol-binding to PFO [73] and it was revealed that cholesterol concentration threshold required for D434S mutation-containing D4 binding is 20 mol % [66]. This D4^D434S^ mutant, named D4H (D4 with higher affinity), is applicable to visualize cholesterol in the cytosolic leaflets of the PM by expression in cytosol [66]. Cytosolically-expressed mCherry-D4H successfully localized in the cytosolic leaflets of the PM in CHO, Raw, and Madin-Darby canine kidney (MDCK) cells in a cholesterol dependent manner ([66,67,74]; Figure 4B and Figure 5B). Importantly, mCherry-tagged not-mutated D4 did not localize in the cytosolic leaflets of the PM in CHO cells and relocalized to the leaflets by addition of cholesterol exogenously [66], suggesting that increased affinity of D4H to cholesterol enabled recognition of lower concentrations of cholesterol in the inner PM. A combination of D4 and D4H to visualize cholesterol in the exofacial PM and cytosolic PM, respectively, showed that phosphatidylserine (PtdSer) is essential for retaining of cholesterol in the cytosolic leaflets of the PM [66]. PtdSer is one of the major phospholipids in the cytosolic leaflets of the PM, which accounts for about 20 mol % of total phospholipids in the leaflets [4,75]. A hallmark of PtdSer in the PM is the asymmetric distribution that PtdSer localizes only in the cytosolic leaflets of the PM in non-stimulated cells [75,76]. By depletion of PtdSer from the PM in CHO cells, binding of recombinant EGFP-D4 proteins to the exofacial leaflets of the PM was increased whereas mCherry-D4H dissociated from the cytosolic leaflets of the PM [66]. Importantly, this phenotype was restored by supplementation of exogenous PtdSer and the existence of PtdSer in liposomes did not effect the binding of D4H to cholesterol in vitro [66]. In vitro cholesterol oxidase accessibility assay clearly showed that 1-stearoyl 2-oleoyl PtdSer (SOPS) can specifically protect cholesterol from cholesterol oxidase in liposomes [66], suggesting that SOPS can interact with cholesterol in phospholipid bilayers. A previous study has shown that, in CHO cells, cholesterol and SOPS are enriched in detergent-resistant membrane fractions, generally called lipid raft fractions [77]. EM analysis clearly showed that PtdSer is enriched in caveolae, the cholesterol-rich nanodomains in the PM [78]. Taken together, these observations strongly suggest the existence of nanodomains formed with PtdSer and cholesterol in the cytosolic leaflets of the PM [66,74]. Further analysis using EM and super-resolution microscopy will be required to observe the PtdSer-cholesterol nanodomains in the cytosolic leaflets of the PM. Although D4H is useful to visualize cholesterol in the cytosolic leaflets of cellular membranes in some cell lines, limitation of D4H should be extensively examined by further experiments. Also, it is noted that, according to liposomal experiments, D4H cannot associate with liposomes in which cholesterol concentration is less than 20 mol % [66].

### 2.5. QYDA, YDA, D434A/A463W

To decrease still high threshold for cholesterol-binding to D4H (D4^D434S^), other point mutations were introduced in D4 in addition to D434 [72]. A recent study identified three amino acid residues (Y415, Q433, and A463) in D4, which determine the affinity of D4 to cholesterol [72]. Liu et al., developed new D4 mutants, D434A, D434/A463W, Y415A/D434W/A463W (named YDA), and Y415A/Q433W/D434W/A463W (named YQDA) [72]. Threshold for cholesterol-binding to D434/A463W was approximately 10 mol %, which is lower than D434A (20 mol %) and not-mutated D4 (30 mol %) [72]. Surprisingly, YDA and YQDA were shown to be able to bind to liposomes in which cholesterol concentration is more than 1 mol % [72], indicating that YDA and YQDA can theoretically label cholesterol in cellular membranes with the wide dynamic range. To visualize cholesterol in cellular membranes using these D4 mutants, the solvatochromic fluorophores, acrlylodan (DAN) or NR3, were conjugated on C459 of D4 [72]. Both DAN-D434A and DAN-WT bound to the exofacial leaflets of the PM, however, neither microinjected DAN-D434A nor DAN-D434A/A463W did not localize in the cytosolic leaflets of the PM in HeLa cells ([72]; Figure 4), suggesting that concentration of cholesterol in the cytosolic PM is lower than 10 mol %. As expected, microinjected NR3-YDA and NR3-YQDA localized in the cytosolic leaflets of the PM ([72]; Figure 4B). Critically, binding of DAN-D4 and DAN-D434A to cholesterol in outer PM-mimicking liposomes was not affected by its surrounding phospholipids (e.g., SM, ceramide, fatty acyl chains of phosphatidylcholine) [72]. Similarly, the affinity of NR3-YDA and NR3-YQDA to cholesterol in inner PM-mimicking liposomes was not changed by its surrounding phospholipids (e.g., PtdSer, phosphatidylethanolamine, fatty acyl chains of phosphatidylcholine) [72]. By making use of these series of D4 mutants, the concentration of cholesterol in the exofacial leaflets and the cytosolic leaflets of the PM was calculated in various cell lines (HeLa, HEK293, NIH3T3, CCD 841, and LS174T cells) [72]. In the experiments, firstly, fluorescence of the cholesterol sensors was calibrated by a fluorescence microscopy in a two-photon excitation mode using giant liposomes (GUVs) in which cholesterol concentration is 0–40 mol % [72]. Then, DAN-D434A (or DAN-D434A/A463W) was added to the culture media to label cholesterol in the outer PM and NR3-YDA (or NR3-YQDA) was microinjected into cytosol to visualize cholesterol in the inner PM followed by observation with a fluorescence microscopy and calculation of cholesterol concentration in each leaflet of the PM [72]. The results clearly indicated that cholesterol concentration was much higher in the exofacial leaflets (30–40 mol %) than in cytosolic leaflets (2–5 mol %) in HeLa, HEK293, NIH3T3, CCD 841, and LS174T cells [72]. These data answer a big question that has been asked for a long time, how is the transbilayer distribution of cholesterol in the PM? A previous study using fluorescent cholesterol analogs (DHE and CTL) and their quenching reagents have shown that 60–70 mol % of the exogenously-loaded sterols localized in the cytosolic leaflets of the PM in TRVb cells, a CHO cell lines stably-expressing the human transferrin receptor [33]. This opposite data on transbilayer distribution of cholesterol might result from differences in cell lines and/or methodology for visualization of cholesterol. Quantification of cholesterol in both bilayer of the PM in CHO cells should be examined using DAN-D434A (or DAN-D434A/A463W) and NR3-YDA (or NR3-YQDA). Liu et al., also showed that methyl-beta-cyclodextrin (MβCD) extracted approximately 75% of cholesterol from the outer PM whereas MβCD reduced cholesterol by about 30% in the inner PM in HeLa cells [72]. One benefit of this methodology is that we can monitor the change of transbilayer distribution of cholesterol in the PM in any physiological events. For example, one of the Wnt ligands, Wnt3a, induced redistribution of cholesterol from the exofacial to cytosolic leaflets of the PM [72,79]. Moreover, ABCA1 and ABCG1, ATP-binding cassette (ABC) transporters, were shown to be essential for maintaining of the low cholesterol concentration in the inner PM in HeLa and HEK293 cells [72,80,81]. More cholesterol localized in the cytosolic leaflets of the PM and less cholesterol localized in the exofacial leaflets of the PM by knockdown of ABCA1 or ABCG1 [72]. Importantly, knockdown of cellular cholesterol transport-related molecules such as NPC1, ORP5, and STARD4 did not effect transbilayer distribution of the PM [2,3,9,10,72,82]. Further analysis of mechanisms for the formation of proper transbilayer distribution of cholesterol in the PM and elucidation of its physiological meanings would be important future works by using DAN-D434A (or DAN-D434A/A463W) and NR3-YDA (or NR3-YQDA). It is noted that, in this method, microinjection of NR3-YDA or NR3-YQDA into cells is required to visualize cholesterol in the cytosolic leaflets of the PM, thus, it is worth examining if cytosolically-expressing other fluorophore (e.g., EGFP, mCherry)-labeled YDA or YQDA can also visualize cholesterol in the cytosolic leaflets of the cellular membranes. This vector-based method (e.g., transfection of plasmids, infection of lentivirus carrying the D4-derived cholesterol probes) would be much easier than microinjection and strongly help researchers to study cholesterol biology in cells.

## 3. Future Perspectives

This review focuses on the latest progress of cholesterol probes based on PFO and D4 domains. It is always noted that localization of cholesterol biosensors derived from PFO would be affected by its surrounding environments (e.g., phospholipids, pH) [50,51,60,61], thus in vitro liposomal reconstitution experiments and filipin staining should be required for assessment of the conclusions. However, the D4 mutants that have higher affinity to cholesterol would be robust tools to analyze the characteristics of cholesterol in the great detail by combination of the state-of-the-art microscopic techniques. Especially, observation of D4 mutants-labeled cells using high-resolution electron microscopy, super-resolution microscopy (e.g., STED microscopy, PALM, SIM), fluorescence recovery after photobleaching (FRAP), single particle tracking (SPT), and fluorescence lifetime imaging microscopy (FLIM) system will provide us with novel information of the clustering and dynamics of cholesterol in cellular membranes in addition to the interaction with other phospholipids like SM, PtdSer, and phosphoinositides. Also, as D4 probes can be applicable to live-cell imaging, we can monitor the dynamics of cholesterol distribution in the cytosolic leaflets of cellular membranes during a variety of physiologically-crucial events such as phagocytosis, macropinocytosis, and angiogenesis by expression of D4 probes in cells. Similarly, transbilayer movement of cholesterol in the PM can be monitored using DAN-D434A (or DAN-D434A/A463W) and NR3-YDA (or NR3-YQDA). This excellent system would uncover the novel mechanisms how cholesterol distribution between cytosolic and exofacial leaflets of the PM is regulated. Interestingly, cytosolically-expressed D4H localizes not only PM and endosomes but also small lipid droplets (LDs) in quiescent cells although this probe comes off enlarged LDs by addition of oleic acids exogenously (unpublished data). Thus, D4H would be useful to study the dynamics of LDs from the standpoint of cholesterol localization. Similarly, NR3-YDA and NR3-YDQA microinjected in the cytosol localized in the cytosolic leaflets of intracellular vesicles in addition to the inner PM of HeLa cell [72]. Thus, these probes would be also robust to monitor endosomal cholesterol distribution. Finally, the precise molecular mechanisms for determination of plasmalemmal localization of cholesterol are still unclear. The genetic screen using siRNA library or CRISPR-Cas9 system in D4H or other D4 mutants stably-expressing mammalian cell lines will identify the essential genes for proper localization of cholesterol in the cytosolic leaflets of the PM.

## Abbreviations

The following abbreviations are used in this manuscript:

PFO	Perfringolysin O
D4	domain 4
ER	endoplasmic reticulum
ACAT	acyl-CoA cholesterol acyl transferase
LD	lipid droplet
LDL	low-density lipoprotein
MVBs	multivesicular bodies
LEs	late endosomes
NPC1	Niemann-Pick disease, type C1
NPC2	Niemann-Pick disease, type C2
PM	plasma membrane
Res	recycling endosomes
OSBP	oxysterol-binding protein
START	steroidogenic acute regulatory protein-related lipid-transfer
DHE	dehydroergosterol
CTL	cholestatrienol
EM	electron microscopy
EGFP	enhanced green fluorescent protein
PALM	photoactivated localization microscopy
CHO cells	Chinese hamster ovary cells
MDCK cells	Madin-Darby canine kidney cells
PtdSer	phosphatidylserine
SOPS	1-stearoyl 2-oleoyl phosphatidylserine
DAN	acrlylodan
GUVs	giant liposomes
MβCD	methyl-beta-cyclodextrin
ABC transporters	ATP-binding cassette transporters
ORP5	oxysterol-binding protein-related protein 5
STARD4	steroidogenic acute regulatory protein-related lipid-transfer protein 4
STED	stimulated emission depletion
SIM	structured illumination microscopy
FRAP	fluorescence recovery after photobleaching
SPT	single particle tracking
FLIM	fluorescence lifetime imaging microscopy
